# Influenza in Children

**Published:** 1898-01-22

**Authors:** 


### MEDICAL PROGRESS AND HOSPITAL CLINICS.

[*The Editor will be glad to receive offers of co-operation and contributions from members of the profession. All letters should be addressed to* THE EDITOR, AT THE OFFICE, 28 & 29, SOUTHAMPTON STREET, STRAND, LONDON, W.C.]

### INFLUENZA IN CHILDREN.

Far less attention has been paid to the influenza of children than to this disease in adults, notwithstanding the fact that at least one-fourth of all influenza cases occurs among children, the mortality amounting to about 20 or 30 per cent. of all such cases. We possess, however, valuable works on influenza in children by Baginsky, Bäumler, Carstens, Fuchs, Fürst, Hagenbach, and Herzog. In addition hereto we have learned a great deal concerning influenza in new-born infants from the researches of Warrington, Townsend, Strassmann, and others.

When a child has an attack of influenza, the process is, as a rule, much less sudden than is the case with adults. It becomes gradually dull, apathetic, and weak, with accompanying catarrhal symptoms. The initial stage begins with an atypical fever and with tendency to rigors. Sometimes there are naso-pharyngeal catarrh, dyspnœa, frequently injection of the conjunctiva and copious secretion of tears; with older children headache, convulsions, and sleepiness; constipation is common.

Should the case be unattended with complication, under careful unremitting treatment with rest in bed, there will be a complete abatement of the symptoms in four or five days. The temperature returns completely and permanently to the normal; and carelessness alone can bring about a relapse. The secretion of the larynx and the bronchial tubes becomes more fluid, the cough loosens, the bronchioles and the lungs remain free. In eight or ten days the period of convalescence is over. Thus the prognosis is by no means unfavourable for simple, uncomplicated cases; a fatal termination can only take place in children whose constitutions are already much enfeebled. Very dangerous, however, are complications involving capillary bronchitis, as they sometimes set in almost unnoticed. This is not infrequently followed by pneumonia, pulmonary œdema, or cardiac weakness. Should the lungs be considerably involved, the prognosis is, of course, much less favourable. Happily, however, this complication can be avoided if the patient is at once put to bed and kept there for six or eight days after convalescence. To delay a suitable treatment too long, or to end it too soon, is alike injurious.

It will thus be seen that the appearance of influenza in children is by no means unattended with danger in all cases, even where suitable treatment and careful nursing have been adopted from the beginning. Although it is thought that the specific cause of influenza has been found in the secretion of the mucous membrane, we are still without any specific remedy against this micro-organism. Indeed, the infectious principle of the influenza catarrh, where possibly the secretion spreads the contagion, is in all probability of a heterogeneous nature. It is also probable that in many children the catarrhal loosening of the epithelical cells forms a favourable bed for the microbes, so that an ordinary catarrh may predispose the patient to an attack of influenza, a fact which is of great prophylactic importance. There is no very definite boundary between the two types of catarrh. Children should, therefore, not only be kept carefully away from diseased persons, but their mucous linings should also, by carefully nursing, be kept in a healthy condition, capable of resisting infection.

With regard to the treatment for influenza in children, specific remedies have been sought which would act directly on the disease. The children have in some instances been benefited by the use of quinine (Roussy, Mosse, Sansom). In other cases, again, salophen has proved efficacious (Drews, Claar, Hennig). Some authorities have given salicylate of sodium, phenacetin, and lactophenin with more or less success. A remedy which, acting almost with the certainty of a specific, may be found in salipyrin, which not only weakens the micrococci and alleviates the catarrhal symptoms, but also clearly diminishes the tendency of the streptococci to invade the lungs and the kidneys. The fever, headache, and prostration generally disappear in about two days, without any deleterious consequences, if the salipyrin is given at once and in not too small doses. A dose, which should be given three times daily, consists of:—

| | | | | |
|---|---|---|---|---|
| 4 grains | for a child | 2— 5 | years of age. |
| 8 | " | " | 6—10 | " |
| 16 | " | " | 11—14 | " |

It may be followed by a cup of tea, to which may be added a little lemon juice and sugar. After two days the dose need only to be given twice daily; it should, however, be continued for about four days, even when there is no trace of fever. The great advantage of this drug lies in the fact, that it has not only absolutely no attendant deleterious effects upon the heart, the central nerve system, and the kidneys, but that, on the other hand, it effectually checks a serious attack of influenza, if given on the very first signs of the disease showing themselves. This has been demonstrated by Bekens, Fürst, Hennig, Moncorvo, Von Mosengeil, Müller, and others. Coryza must be symptomatically treated, and, if possible, suppressed with menthol inhalation. Spasmodic laryngo-bronchial-catarrh, as also any tendency to whooping cough, are effectually treated with salipyrin. Middle-ear affections can be avoided by careful treatment of the naso-pharyngeal-catarrh.

After recovering from influenza it is absolutely necessary that the children should be guarded against fresh attacks by daily frictions of the skin, and by disinfecting the mucous membrane of the mouth, nose, and pharynx with cool, antiseptic washes; they should also be protected from any liability to catch fresh cold. They should be strictly kept away from persons affected with influenza, as relapses are by no means infrequent. This after-treatment should never be omitted, as the children are very susceptible to second infection.

